# 1247. Primary Care Teams in Veterans Health Administration Have High Knowledge of Universal HCV Screening But Favor System-Level Interventions to Close Care Gaps

**DOI:** 10.1093/ofid/ofac492.1078

**Published:** 2022-12-15

**Authors:** Arpan Patel, Neaka Mohtashemi, Yash Motwani, Chi-hong Tseng, Jenna Kawamoto, Phillip Chen, Manuel A Celedon, Jihane Benhammou, Tien Dong, Folasade May, Matthew B Goetz, Debika Bhattacharya

**Affiliations:** University of California, Los Angeles and VA Greater Los Angeles Healthcare System, Los Angeles, California; UCLA and Greater Los Angeles VA, Los Angeles, California; UCLA Health, Los Angeles, California; UCLA, los angeles, California; VA Greater Los Angeles Healthcare System, Los Angeles, California; UCLA, LOS ANGELES, California; Assistant Clinical Professor of Emergency Medicine David Geffen School of Medicine at UCLA, Los Angeles, California; UCLA and Greater Los Angeles VA, Los Angeles, California; University of California, Los Angeles, Los Angeles, California; UCLA HEALTH, Los Angeles, California; VA Greater Los Angeles Healthcare System, Los Angeles, California; University of California, Los Angeles, Greater Los Angeles Veteran Affairs, Los Angeles, CA

## Abstract

**Background:**

In 2020, the United States Preventive Services Taskforce recommended one-time Hepatitis C virus (HCV) screening for all adults aged 18-79. As the largest provider of HCV treatment in the US the Veterans Health Administration (VA) is committed to implementing these recommendations; thus, understanding perspectives of primary care teams towards this is critical.

**Methods:**

We disseminated a 24-item online survey to PCPs at the VA Greater Los Angeles Healthcare System (VAGLAHCS), serving 1.5 million Veterans within an area that spans five counties. Our sample included physicians, pharmacists, nurse practitioners, and physician assistants on primary care teams. Our survey contained questions on demographics, provider knowledge of HCV testing, practice patterns and beliefs, and preferences for new screening tools. Descriptive statistics were completed and we compared item responses across demographics. Hypothesis testing was performed using Wilcoxon rank sum test for continuous variables and Fisher’s exact or Chi-square tests for categorical variables.

**Results:**

In total, 107 PCPs responded to the survey (107/385=28% response rate), with 100 complete responses. Most respondents were women (63%) with median age of 42 years, physicians (53%), Internal Medicine-trained (47%), and of Asian descent (45%). A majority (59%) were aware of new screening recommendations. Most PCPs reported confidence (71%), intent (77%), and commitment towards (82%) HCV screening. Only 38% reported remembering to screen Veterans regularly. Physicians were most aware of new recommendations (73%), compared to pharmacists (55%) and nurse practitioners (42%). The most strongly preferred strategies to enhance screening included automatic test orders during physician visits (71%), automatic electronic-consults (68%), clinician reminders for HCV screening (57%), and opt-out phlebotomy (48%).

**Conclusion:**

In a sample of providers within a large and diverse VA, 3 in 5 PCPs were aware of new universal HCV screening recommendations. There is high motivation among PCPs to perform screening, but forgetting to screen is still common. Several strategies to augment universal HCV screening were acceptable towards PCPs.

**Disclosures:**

**Debika Bhattacharya, MD, MSc**, Gilead: Grant/Research Support|Regeneron: Grant/Research Support.

